# Repeated Adaptive Introgression at a Gene under Multiallelic Balancing Selection

**DOI:** 10.1371/journal.pgen.1000168

**Published:** 2008-08-29

**Authors:** Vincent Castric, Jesper Bechsgaard, Mikkel H. Schierup, Xavier Vekemans

**Affiliations:** 1 Université des Sciences et Technologies de Lille 1, Laboratoire Génétique et Evolution des Populations Végétales, CNRS UMR 8016, France; 2 Ecology and Genetics, Institute of Biological Sciences, University of Aarhus, Denmark; University of Chicago, United States of America

## Abstract

Recently diverged species typically have incomplete reproductive barriers, allowing introgression of genetic material from one species into the genomic background of the other. The role of natural selection in preventing or promoting introgression remains contentious. Because of genomic co-adaptation, some chromosomal fragments are expected to be selected against in the new background and resist introgression. In contrast, natural selection should favor introgression for alleles at genes evolving under multi-allelic balancing selection, such as the MHC in vertebrates, disease resistance, or self-incompatibility genes in plants. Here, we test the prediction that negative, frequency-dependent selection on alleles at the multi-allelic gene controlling pistil self-incompatibility specificity in two closely related species, *Arabidopsis halleri* and *A. lyrata*, caused introgression at this locus at a higher rate than the genomic background. Polymorphism at this gene is largely shared, and we have identified 18 pairs of S-alleles that are only slightly divergent between the two species. For these pairs of S-alleles, divergence at four-fold degenerate sites (*K = *0.0193) is about four times lower than the genomic background (*K = *0.0743). We demonstrate that this difference cannot be explained by differences in effective population size between the two types of loci. Rather, our data are most consistent with a five-fold increase of introgression rates for S-alleles as compared to the genomic background, making this study the first documented example of adaptive introgression facilitated by balancing selection. We suggest that this process plays an important role in the maintenance of high allelic diversity and divergence at the S-locus in flowering plant families. Because genes under balancing selection are expected to be among the last to stop introgressing, their comparison in closely related species provides a lower-bound estimate of the time since the species stopped forming fertile hybrids, thereby complementing the average portrait of divergence between species provided by genomic data.

## Author Summary

The role of natural selection in promoting or preventing genomic divergence between nascent species remains highly debated. As long as reproductive barriers remain incomplete, genetic material from one species is indeed exposed to natural selection into the genomic background of the other species. In some cases, genomic co-adaptations developing independently in each species are believed to select against such transfers. Yet, theory predicts that the transfer of some chromosomal fragments may be favored by natural selection. In particular, this should occur for alleles at genes evolving under a particular form of natural selection, i.e., multi-allelic balancing selection. We test this prediction using two closely related *Arabidopsis* species, and find a four-fold lower divergence at alleles at the gene controlling pistil self-incompatibility specificity than at the genomic background. We conclude that alleles at this gene have been transferred more readily between the two species than the genomic background. We suggest that natural selection may efficiently allow the maintenance of high allelic diversity and divergence across many species at S-loci as well as at all other loci under multi-allelic balancing selection, such as the MHC in vertebrates or disease resistance genes in plants.

## Introduction

The genomes of incipient species diverge at heterogeneous rates, and recently diverged model species are key systems to investigate the causes of this heterogeneity [1]–[3]. Hybridization followed by introgression between recently diverged plant and animal species with incomplete reproductive barriers is one of the main processes generating the genomic heterogeneity in species divergence [4]. Indeed, some regions appear to be crossing the species barriers more readily than the genomic background (in Helianthus [5], Anopheles [6], Quercus [7], Mytilus [8], Mus [9] and Drosophila [10]). Although much of this heterogeneity may be accounted for by stochasticity of the genetic drift process, natural selection may also play an important role. In particular, because introgressive hybridization brings genetic material from one species into the co-adapted background of another species, some chromosomal fragments are expected to be selected against and resist introgression [11].

On the other hand, selection can also promote introgression when a transferred chromosome fragment is advantageous in the recipient species. In such a situation, introgression can potentially mediate the transfer of adaptations. Examples of adaptive introgression involving the transfer of transgenes conferring adaptations such as herbicide or insect resistance *via* hybridization with close relatives of crop species [12] have been documented, but other examples in natural populations are strikingly rare [13]. In the Louisiana Iris species complex for instance, detailed experimental studies provided support for the transfer of adaptations (flood and shade tolerance) between *Iris fulva* and *I. hexagona* [14]. In Helianthus, a recent experimental study reported that herbivore resistance traits have introgressed from *Heliantus debilis* to *H. annuus,* thereby increasing adaptation of their naturally occurring hybrid *H. annuus taxanus* [15]. All these documented examples are thus associated with strong directional selection for adaptive traits recently evolved in one of the species and then transmitted horizontally. Theory predicts that adaptive introgression should also be a general property of alleles at genes evolving under multi-allelic balancing selection, such as the vertebrate MHC system, plant disease resistance or self-incompatibility (SI) genes [16]. In these systems, rare alleles enjoy a strong selective advantage [17]. Assuming that a given allele is absent from one of two related species, introgression of this allele would then be as strongly favored as a new allele arising by mutation, unless this is impeded by linked genes that are not well adapted to the recipient species. Thus, in multi-allelic systems evolving under balancing selection, repeated exchanges of alleles promoted by adaptive introgression may be expected between closely related species, as long as fertile hybrids can be formed. Therefore, in the course of evolution of strong reproductive isolation between incipient species, such genomic regions should be among the last to stop introgressing.

In this study, we test whether multi-allelic balancing selection mediates introgression between closely related species. We do this by contrasting divergence of a portion of the gene controlling self-incompatibility specificity (*SRK*) with the background level of genomic divergence in two closely related plant species. The study system consists of two closely related *Arabidopsis* species, *A. lyrata* and *A. halleri*, whose genomes diverged approximately 2 million years ago [18]. The two species have overlapping distributions in Northern Europe [19] and relatively recent introgression has been demonstrated for a small fraction of nuclear genes [20]. SI prevents self-fertilization and some matings among relatives through recognition and rejection of pollen expressing identical specificity. Molecular and genetic analyses of the self-incompatibility locus (S-locus) in *A. lyrata* and *A. halleri* identified many specificities, and the *SRK* sequences often form monophyletic pairs of high sequence similarity, each of which probably represent the same SI specificity in the two species derived from one specificity in their common ancestor. We refer to these pairs as trans-specifically shared pairs of S-alleles. We use divergence at fourfold degenerate sites between alleles within trans-specifically shared pairs to estimate the divergence corresponding to the time of the last introgression event for S-alleles between the two species, and we find that introgression has occurred at a higher rate or continued over more extended periods of time at the S-locus than at the rest of the nuclear genome.

## Results

### Extent of trans-Specific Allele Sharing at *SRK*

**Figure 1:**
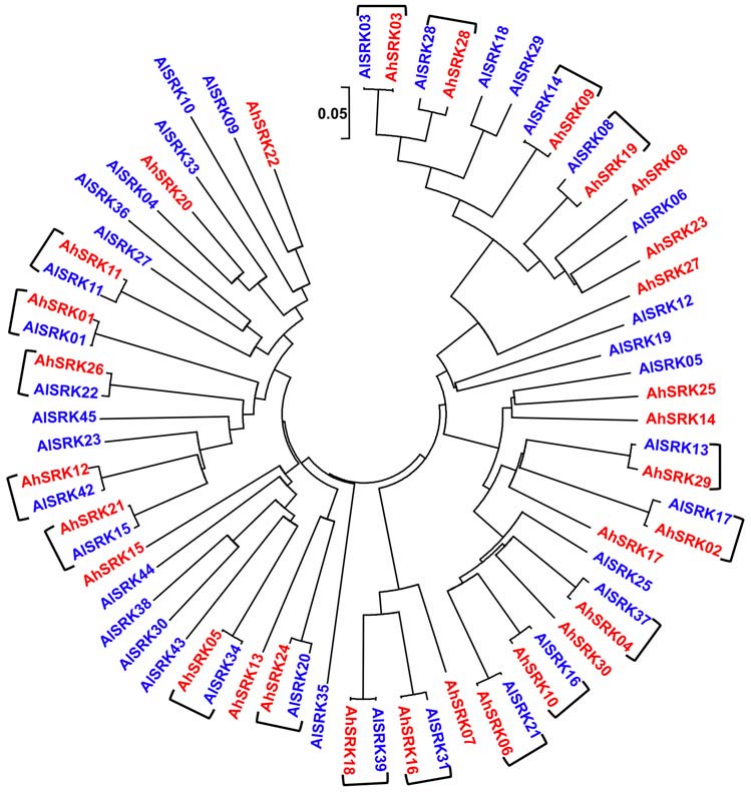
Phylogeny of the 68 *SRK* sequences of *A. lyrata* and *A. halleri.* The phylogeny was obtained by the neighbour-joining method on pairwise proportion of nucleotide divergence after Jukes-Cantor's correction. Brackets indicate interspecific pairs of sequences assumed to represent “trans-specifically shared S-alleles”, *i.e.* alleles assumed to have evolved from a single S-allele in the direct ancestor of *A. lyrata* and *A. halleri*.

**Figure 2:**
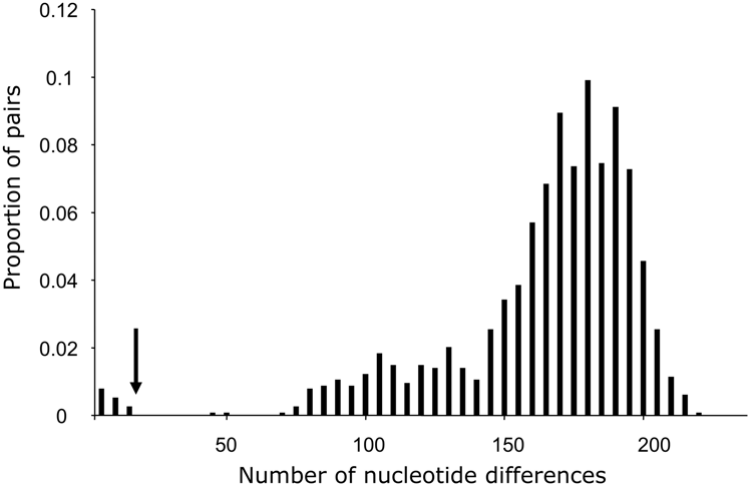
Distribution of the number of pairwise nucleotide differences for *SRK* sequences in interspecific comparisons between *A. halleri* and *A. lyrata*. Note the distinct peak of highly similar sequences observed. The vertical arrow represents the chosen threshold to define “trans-specifically shared” pairs of sequences (≤12 nucleotide differences). Note also that the two pairs of sequences with intermediate nucleotide differences (45 between *AlSRK03* and *AhSRK28*, and 50 between *AlSRK28* and *AhSRK03*) cannot represent trans-specifically shared S-alleles because they are not monophyletic (see Figure 1).

Our species-wide survey of sequence diversity reveals that a large fraction of alleles at the pistil self-incompatibility specificity-determining gene *SRK* (S-locus receptor kinase) are trans-specifically shared between the two species (Figure 1). Overall, we find 30 sets of *SRK* sequences in *A. halleri* and 38 sets of *SRK* sequences in *A. lyrata.* As is typical for S-alleles [21], the sequences fall into sets of nearly identical ones (presumably representing the same specificity, [21]–[23]) and ones with many differences from all other sequences (presumably representing functionally distinct specificities), with the most similar pairs within *A. halleri* and *A. lyrata* showing 44 and 51 differences, respectively, over a total of about 570 nucleotides. We then compared nucleotide sequences between S-alleles from the two species and find that the mismatch distribution (Figure 2) is clearly bimodal. Most comparisons are in line with intraspecific comparisons and range between 45 and 218 differences over a total of about 570 nucleotides (see also Figure S5), but the distribution shows a distinct set of 18 highly similar interspecific pairs of sequences (indicated by brackets in Figure 1) with at most 12 nucleotide differences. The numbers of non-synonymous differences within the 18 highly similar pairs of S-alleles ranged from 0 to 9 over a total of 380 non-synonymous sites. These sequences are more similar than pairs of alleles known to have retained the same specificity when comparing the closely related *Brassica oleracea* and *B. rapa* [24]–[27]. Even if these sequences currently occur in two different (but closely related) species, we therefore hypothesize that these pairs have retained identical specificity. We refer to these 18 pairs of S-alleles as “trans-specifically shared” pairs of alleles and note that they represent 60% and 47% of S-alleles found to date in *A. halleri* and *A. lyrata*, respectively. Two of these pairs (*AlSRK37*/*AhSRK04* and *AlSRK16*/*AhSRK10*) were previously identified and shown additionally to be shared trans-specifically with *A. thaliana* [28]. Phylogenetic reconstructions show that both synonymous (Figure S2) and non-synonymous (Figure S3) differences are strikingly low within trans-specifically shared pairs and high among pairs. Note that, by definition, *SRK* alleles we consider as trans-specific pairs are determined based on those S allele pairs that have the fewest differences, so the procedure could potentially lead to ascertainment bias. Yet, close examination of the next best candidates (*AhSRK03/AlSRK28*, *AhSRK28/AlSRK03*, *AhSRK23/AlSRK06* and *AhSRK20/AlSRK04,* Figure 1) suggests that none of these pairs is likely to represent pairs of trans-specifically shared alleles (detailed arguments are presented in Text S1).

### Divergence within trans-Specifically Shared Pairs of S-Alleles

Within *SRK*, several hypervariable (HV) regions have been identified in the domain responsible for binding the pollen protein (S-domain) and shown to be targets of positive selection, suggesting they are involved in determination of specificity [29],[30]. Accordingly, HV regions from different specificities within species typically show an excess of non-synonymous substitutions [29],[31],[32]. In sharp contrast, we find that as compared to synonymous differences, non-synonymous differences are relatively less frequent in HV regions than in non-HV regions (on average 0.7 and 2.3 differences in HV and non-HV regions respectively for non-synonymous differences, *versus* 1.1 and 1.6 differences respectively for synonymous differences, Table 1). This contrast is significant by Fisher's exact test of independence (odds ratio = 2.5, *p* = 0.029), suggesting that sequence pairs that putatively encode the same specificity tend to have similar HV region sequences for non-synonymous sites, but might differ at synonymous sites in these regions, whereas other regions may differ at both types of sites.

**Table 1:** Divergence between *Arabidopsis halleri* and *A. lyrata* at trans-specifically shared *SRK* alleles at synonymous (*K*_S_), non synonymous (*K*_A_) and fourfold degenerate sites (*K*_4fold_).

		Coding sequence length	Nucleotides in HV	Synonymous differences		Non-synonymous differences		Nucleotide divergence		
				in HV	not in HV	in HV	not in HV	*K*_S_	*K*_A_	*K*_4fold_
AlSRK01	AhSRK01	578	124	0	1	1	2	0.0080	0.0045	0
AlSRK03	AhSRK03	598	124	0	0	0	1	0	0.0021	0
AlSRK08	AhSRK19	593	124	3	4	1	3	0.0581	0.0086	0.0969
AlSRK11	AhSRK11	565	122	1	2	2	7	0.0241	0.0185	0.0423
AlSRK13	AhSRK29	537	91	2	3	1	2	0.0444	0.0072	0.0330
AlSRK14	AhSRK09	598	124	0	0	0	3	0	0.0064	0
AlSRK15	AhSRK21	590	124	1	3	1	0	0.0331	0.0022	0.0163
AlSRK16	AhSRK10	592	124	1	2	0	2	0.0158	0.0022	0
AlSRK17	AhSRK02	557	118	2	1	1	3	0.0251	0.0093	0.0160
AlSRK20	AhSRK24	551	106	1	4	0	4	0.0446	0.0093	0.0347
AlSRK21	AhSRK06	516	124	2	1	0	0	0.0260	0	0
AlSRK22	AhSRK26	568	122	0	1	0	7	0.0081	0.0137	0
AlSRK28	AhSRK28	548	124	3	0	0	0	0.0258	0	0.0300
AlSRK31	AhSRK16	509	91	0	2	1	2	0.0194	0.0075	0.0174
AlSRK34	AhSRK05	539	111	0	1	0	2	0.0084	0.0048	0.0163
AlSRK37	AhSRK04	592	124	3	1	1	2	0.0315	0.0087	0.0287
AlSRK39	AhSRK18	573	124	0	0	0	0	0	0	0
AlSRK42	AhSRK12	552	124	1	2	3	2	0.0261	0.0116	0.0163
**Average**		**569**	**118**	**1.1**	**1.6**	**0.7**	**2.3**	**0.0221**	**0.0065**	**0.0193**

All estimates were Jukes & Cantor corrected. HV refers to hypervariable regions as defined by Nishio and Kusaba [60].

If introgression occurs, then divergence might also be affected by the dominance of the S-alleles. Indeed, complex patterns of dominance relationships generally occur among alleles in sporophytic SI systems [33] and Billiard et al. [34] reported asymmetric selective pressures for dominant and recessive S-alleles because rare dominant S-alleles will tend to express their specificity more often than rare recessive ones (a process similar to “Haldane's sieve”-the bias against the establishment of recessive beneficial mutations [35],[36]). Hypothesizing that the introgression rate thus differs between dominant and recessive S-alleles, we tested for an effect of dominance on divergence between the two species. The range of variation observed for nucleotide differences across pairs of trans-specifically shared S-alleles cannot be explained fully by the stochasticity of the substitution process (Fisher's dispersion index = 2.03, P = 0.0103), but there was no obvious relationship between number of nucleotide differences and level of dominance of the S-alleles, as inferred from the phylogeny of alleles as suggested by [37]. Thus, we find no evidence that dominance affects S-allele divergence between the two species.

### Comparison of Introgression Rate between S-Locus and Genomic Background

To test whether balancing selection resulted in adaptive introgression of S-alleles between the two species, we compared levels of divergence at fourfold degenerate sites between trans-specifically shared S-alleles with that of the genomic background, estimated from twelve unlinked control genes and two S-gene family members. These two sets of control genes give similar mean values of divergence (*K*_4fold_ = 0.0743 and *K*_4fold_ = 0.0904, respectively, Table 2), which are about four times higher than the average for trans-specifically shared pairs of S-alleles (*K*_4fold_ = 0.0193, Table 1).

**Table 2:** Divergence between *Arabidopsis halleri* and *A. lyrata* at reference genes and members of the S-gene family at fourfold degenerate sites (*K*_4fold_).

		Coding sequence length	Number of sequences analysed		*K*_4fold_	References
			*A. lyrata*	*A. halleri*		
Genomic background	CAD	956	8	8	0.0483	20
	CHI	264	10	10	0.1468	20
	CHS	1177	12	11	0.0881	20
	DFR	346	10	8	0.0610	20
	F3H	450	10	10	0.1223	20
	FAH1	1054	10	8	0.0739	20
	GS	906	14	12	0.0861	20
	MAML	388	12	11	0.0312	20
	CAUL	246	18	36	0.0184	39, 40
	HAT4	340	19	34	0.0531	39, 40
	ScADH	515	27	34	0.0323	39, 40
	Aly9	443	12	28	0.1296	39, 40
	**Average**				**0.0743**	
S-gene family	Aly10.1	936	1	1	0.1043	this study
	Aly10.2	466	1	1	0.0765	this study
	**Average**				**0.0904**	

All estimates were Jukes & Cantor corrected.

*: Note that Aly 9 is a member of the S-domain gene family, but polymorphism data for this gene was used here to increase the genomic background dataset.

**Figure 3:**
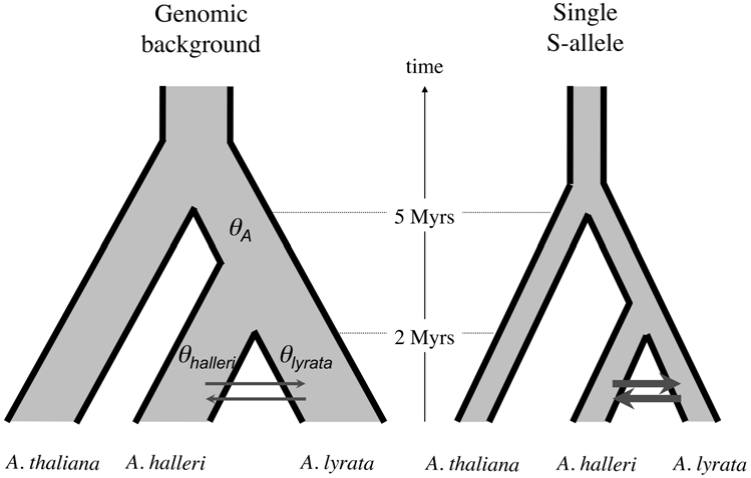
Divergence process between *Arabidopsis lyrata*, *A. halleri* and *A. thaliana* at unlinked genes (genomic background) and trans-specifically shared pairs of S-alleles. Divergence times were taken from Koch et al. [18],[58]. *θ_lyrata_*, *θ_halleri_* and *θ_A_*, refer to polymorphism (*θ = *4*Nμ·* in *A. lyrata*, *A. halleri* and their common ancestor. As compared to unlinked genes, divergence between trans-specifically shared S-alleles is affected by two confounding factors: (1) lower effective population size than the genomic background reducing coalescence time in the ancestral species, and (2) expected higher introgression as represented by thicker dark grey arrows.

**Figure 4:**
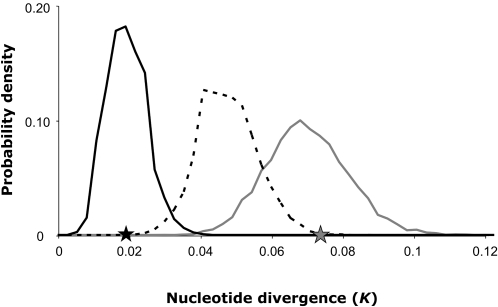
Predicted nucleotide divergence between *A. halleri* and *A. lyrata* for the genomic background (grey line), S-alleles with the same rate of introgression as the genomic background (dotted line) and S-alleles with 5-fold increased rate of introgression relative to the genomic background (black line). 10,000 coalescent simulations were performed for each case using maximum likelihood parameter estimates obtained under the “isolation with migration” model, except for the dotted line, where the 2.5% low ancestral population size estimate was used in order to be conservative. Observed nucleotide divergence for the genomic background and S-alleles are represented by grey and black stars on the x-axis, respectively.

Because a large number of S-alleles are actively maintained within species by balancing selection, each S-allele has individually a small effective population size [21]. Thus, estimates of divergence for S-alleles and reference genes cannot be compared directly because of differences in effective population sizes (Figure 3). To take this into account, we used coalescent simulations to test whether our data are compatible with a null model of speciation (the “isolation with migration” model of Nielsen and Wakeley, [38]) that assumes identical introgression rate for S-alleles and the genomic background. Under this model, we first used previously published species-wide polymorphism data in *A. halleri* and *A. lyrata* from [20],[39],[40] to estimate rates of introgression, splitting time *t* as well as *θ_A_* = 4*N_A_μ*, where *N_A_* is the effective population size in their common ancestor and *μ* the substitution rate. The maximum likelihood estimates for directional rates of introgression are *m_hal→lyr_* = 2.775×10^−7^, *m_lyr→hal_* = 2.912×10^−7^, and *θ_A_* = 1.7975 (Table 3). The *t* estimate is 2,533,980 years [1,307,952–5,166,833], which is entirely consistent with the previous 2 Myrs estimate by Koch & Matschinger [18]. All estimates converge satisfactorily based on 10 replicate runs with different random seeds. To single out the *N_A_* estimate, we then used *A. thaliana* as outgroup to obtain a substitution rate at fourfold degenerate sites of *μ* = 1.296×10^−8^ substitutions per nucleotide per year ([9.218×10^−9^–1.781×10^−8^] as 95% credible interval). The resulting estimate for *N_A_* is 253,892 with [13,772–663,510] as 95% credible interval. Based on these parameters, we then simulated the evolution of two species exchanging migrants at the rate estimated above. The simulations were entirely consistent with the data for the genomic background (*K* = 0.0678 [0.0423–0.0955], Figure 4). In sharp contrast, conservatively assuming a reduction of effective population size for S-alleles by a factor 50 (as expected if 50 different S-alleles segregate in each species) only led to a modest reduction in divergence (*K* = 0.0465, Figure 4), whose 95% credible interval [0.0305–0.0640] did not comprise the observed value for *K* (*K*_4fold = _0.0193). Hence, the data are not consistent with equal introgression for S-alleles and the genomic background. This result is robust to the conservative use of the lower boundary of the 95%CI for either *N*_A_ or *t*. Increasing the rates of introgression for S-alleles led to a sharp reduction in divergence between *A. halleri* and *A. lyrata.* The simulations best fitted the data when the directional rates of introgression were empirically increased for S-alleles by a factor 5, with divergence value closely approaching the observed data (*K* = 0.0182, Figure 4). A simpler analysis also confirmed that average net interspecific divergence [41] for S-alleles was lower than that at the genomic background (Text S1).

**Table 3:** Estimates of *θ = *4*Nμ*, effective population sizes, splitting time and rates of introgression using the isolation with migration model [38].

Parameter	ML estimate	95% CI
Common ancestor		
* θ_A_*	1.7975	0.0975–4.6975
* N_A_*	253,892	13,772–663,510
*A. lyrata*		
* θ_lyrata_*	1.2225	0.7635–1.8405
* N_lyrata_*	172,675	107,842–259,966
*A. halleri*		
* θ_halleri_*	0.7785	0.4635–1.2195
* N_halleri_*	109,961	65,468–172,251
Splitting time		
* t* (years)	2,533,980	1,307,952–5,166,833
Rates of introgression		
* m_hal→lyr_*	2.775×10^−7^	5.186×10^−7^–7.510×10^−7^
* m_lyr→hal_*	2.912 ×10^−7^	2.035×10^−7^–1.059×10^−7^

*N_A = _*Effective population size in the common ancestor of *A. lyrata* and *A. halleri.*

*N_lyrata = _*Effective population size in *A. lyrata*.

*N_halleri = _*Effective population size in *A. halleri*.

*m_hal→lyr_ = *Rate at which genes come into *A. lyrata* from *A. halleri* as time moves forward.

*m_lyr→hal_* = Rate at which genes come into *A. halleri* from *A. lyrata* as time moves forward.

For three pairs of S-alleles (*AlSRK01/AhSRK01*, *AlSRK34/AhSRK05*, *AlSRK37/AhSRK04*) we also surveyed intra-allelic variation in at least 10 copies from each species. We found very little diversity among allelic copies within each surveyed allele in each species (average synonymous diversity = 0.0064, data not shown) in accordance with their low expected effective population sizes. We examined the sequences for shared polymorphisms, and found none in any of these S-allele pairs. This suggests old and infrequent, rather than recent, introgression events since the separation of *A. lyrata* and *A. halleri*. Moreover, the estimated divergence among pairs of S-alleles was more heterogeneous than expected based on the Poisson distribution, suggesting that the last introgression event occurred at different times for different alleles.

## Discussion

### Impact of Founder Events at Speciation

The possibility of introgression of S-alleles may have important consequences for the extent of allelic diversity maintained within self-incompatible species. If introgression occurs, hybridizing species effectively share a common pool of S-alleles. If hybridization is restricted, the two species together can maintain more S-alleles than each species individually [42]. Such a process could be especially important in the first stages of the split because reproductive barriers may then be more leaky, and also because allelic diversity at the S-locus within incipient species may be decreased if founding events were associated with speciation. This process could be responsible for maintaining many highly divergent allelic lineages at the S locus within plant families, where trans-generic sharing of allelic lineages seems to be the rule, and loss of ancestral allelic lineages through strong bottlenecks within particular genera the exception, as has been described in the Solanaceae [43].

It can therefore be misleading to use a species' extant number of lineages at a gene under balancing selection to estimate the minimum population size at speciation. For instance, using polymorphism data for MHC in humans, Takahata [44] predicted that the number of breeding individuals in the human lineage could not be as small as 50-100 at any time of its evolutionary history, assuming two extant ancestral allelic lineages at HLA-B. According to our hypothesis of adaptive introgression mediated by balancing selection, variation can be efficiently “rescued”, and stronger founder events at speciation would still be compatible with extant variation at HLA-B, if some interbreeding occurred with the chimpanzee lineage after the split. Although identifying the functional types of alleles may not be simple in that case (and recombination may confine the effect of balancing selection to a small region around the selected sites themselves), a detailed analysis of MHC alleles in the great apes would be of great interest to survey whether adaptive introgression mediated by balancing selection has indeed occurred in primates.

### Shared Chloroplast Haplotypes: Distinguishing between Introgression and Ancestral Polymorphism

A recent study by Koch and Matschinger [18] reported that, whereas *A. lyrata* and *A. halleri* were well separated in phylogenetic trees based on the nuclear encoded ITS region, several cpDNA haplotypes are shared between both species [18]. This was interpreted as ancestral polymorphism segregating for the chloroplast but not the nucleus. However, this interpretation is at odds with the smaller effective population size expected for the chloroplast (approximately 1/2 for hermaphroditic species, [45]) and the consequent low expected variability. Indeed most studies in plants have found low sequence diversity for chloroplast genes, taking into account their low mutation rate [46], and also stronger differentiation among populations for chloroplast than nuclear markers [47]. In line with our results from S-alleles, we suggest the alternative interpretation that introgression occurred more readily for the chloroplast than nuclear genes, as has been reported in several instances (e.g. [48],[49]). The haplotype network of chloroplast sequences reported by Koch and Matschinger [18] also showed greater sharing of more basal haplotypes, suggesting that chloroplast introgression has become less common in recent times.

### Evolution of New Specificities of Self-Incompatibility Genes

Our results also shed light on the evolution of self-incompatibility specificities. Indeed, our data strongly suggest that purifying selection prevents the substitution of non-synonymous differences within HV regions, supporting a role for these regions in determining specificity. More specifically, the strength of purifying selection seems higher on the HV regions than on the rest of the sequence, and this could be related to strong selection against mutations altering specificities. Mechanisms selecting against mutant S-alleles with altered pistil specificities have been discussed by Uyenoyama et al. [50].

Inter-species exchanges of S-alleles may, however, be important in the evolution of new specificities. Chookajorn et al. [51] suggested that new specificities could evolve if sufficient variation could be maintained within the pollen (or pistil) S gene for enough time to allow variants of the other gene to co-evolve with them. Due to the small effective population size of individual S-alleles, this hypothesis requires population structure with very limited migration [16]. Speciation with some introgression of S-alleles leads to precisely the strongly subdivided population needed for this mechanism to work. Under this hypothesis, two alleles could slowly evolve to different specificities in two isolated species and then add to the number of S-alleles in each species after reciprocal introgression. Data testing the specificities of sequence pairs in the two species that differ at few amino acids might help determine whether new specificities have indeed arisen in one species or the other since they split.

## Material and Methods

### Extent of trans-Specific Allele Sharing at *SRK*

We surveyed sequence diversity at *SRK* in two species-wide samples in *A. halleri* and *A. lyrata* over a total of about 570 nucleotides from the 3′ end of the S-domain using the strategy detailed in [31]. We identified and sequenced five and eight new putative S-alleles in *A. halleri* and *A. lyrata*, respectively. Overall, we analyzed 30 *SRK* sequences in *A. halleri* and 38 sequences in *A. lyrata*. In each case, the nucleotide sequence was obtained as a consensus over three independently obtained sequence products. All identified sequences in *A. halleri* and *A. lyrata* were amino-acid translated and aligned by ClustalW in BioEdit 7.0.5 [52] and adjusted by eye. On the overall set of sequences at *SRK*, we used MEGA 4 [53] to reconstruct a phylogeny using the Neighbor-Joining method based on the total number of differences per site or on the number of either synonymous or non-synonymous differences.

### Divergence within trans-Specifically Shared Pairs of S-Alleles

Within each pair of trans-specifically shared sequences at *SRK*, we estimated the number of synonymous nucleotide differences per synonymous site between the *A. halleri* and the *A. lyrata* copy using the method of [54] with MEGA 4. A homogeneous substitution process across all pairs is expected to result in an accumulation of nucleotide differences according to the Poisson distribution. We used Fisher's dispersion index to test whether the distribution of nucleotide differences across trans-specifically shared sequence pairs could be explained by the stochasticity of the substitution process alone. We used Fisher's exact test of independence to test whether synonymous and non-synonymous differences hit HV regions equally frequently.

### Inference on Introgression Patterns at the S-Locus

Background genomic divergence was estimated by the species-wide average nucleotide divergence at fourfold degenerate sites (*K*_4fold_) between the two species for 12 reference genes that had been previously sequenced [20],[39],[40] and two genes that are members of the S-domain gene family (*Aly10.1, Aly10.2*).

To determine whether difference in effective population size and thus coalescence time between S-alleles and genomic background may suffice to explain the low divergence of S-alleles, we applied the isolation with migration model of Nielsen and Wakeley [38] to polymorphism at fourfold degenerate sites in both species for the eleven reference genes plus Aly9 (12 genes in total, see table 2) as implemented in the IM program [38]. We chose to focus on fourfold degenerate sites only because differences in substitution rates have been reported among codon positions [55]. The program DNAsp [56] was used to generate a datafile containing fourfold degenerate sites only. The procedure was run with 10 different random seeds to ensure proper convergence of the six free parameters, *i.e. θ_A_*, *θ_lyrata_*, *θ_halleri_, t, m*_hal→lyr_, *m*_lyr→hal_ (polymorphism *θ* = 4*Nμ* in the common ancestor of *A. halleri* and *A. lyrata*, polymorphism in *A. lyrata*, polymorphism in *A. halleri*, splitting time and the rate at which genes introgressed into *A. lyrata* from *A. halleri* and into *A. halleri* from *A. lyrata* as time moves forward, respectively). The HKY mutation model [57] was used. To single out the *N_A_* estimate, we estimated the average per fourfold degenerate site mutation rate (*μ*) as follows. We used *A. thaliana* as outgroup to estimate the average net nucleotide divergence at fourfold degenerate sites between *A. thaliana* and *A. halleri* and between *A. thaliana* and *A. lyrata* for each reference gene. Assuming that the lineages leading to *A. thaliana* and the common ancestor of *A. lyrata* and *A. halleri* separated 5 million years ago [58], we obtained a mutation rate estimate per site per year for each reference gene. We computed an average mutation rate per site per year (*μ*) by taking the geometric mean over genes. A mutation rate per generation was computed assuming a mean generation time of two years.

The maximum likelihood estimates were then used to simulate divergence between two species isolated since one million generations but still capable of introgression. Ten thousand replicates of pairs of genes with the same number of nucleotides as the real data were performed using SIMCOAL2 [59]. The genomic background divergence was first used to confirm that the simulations parameters were appropriate. We then determined whether the observed divergence for S-alleles was consistent with the overall genomic rate of introgression by simulating the evolution of S-alleles in this system assuming that 50 S-alleles segregate in the species, and thus that the effective population size of each allelic class is reduced by a factor 50. To remain conservative in this analysis, S-alleles were simulated under the 2.5% low boundary of the 95% credible interval for *N_A_* obtained from IM.

Using the maximum likelihood estimate for *N_A_*, we then aimed to determine the extent to which introgression is increased for S-alleles relative to the genomic background. We did so by gradually increasing *m*_hal→lyr_ and *m*_lyr→hal_ for S-alleles by a multiplicative factor from one to ten until the simulated data came close to the observed divergence.

The sequences reported in this paper have been deposited in the GenBank database under accession numbers EU878008- EU878026.

## Supporting Information

Figure S1 Phylogeny of SRK sequences from the species *A. lyrata* (n = 38), *A. halleri* (n = 30) and *Capsella grandiflora* (n = 7, shown in bold). The phylogeny was obtained by the neighbour-joining method on the proportion of amino-acid differences. Brackets indicate the position of two trans-specifically shared pairs of S-alleles between *A. lyrata* and *A. halleri* that are interrupted by the branching of one S-alleles from *C. grandiflora* (thick lines).
(1.16 MB TIF)

Figure S2 Phylogenies of 68 SRK sequences from *A. lyrata* and *A. halleri*. The phylogeny was obtained by the neighbour-joining method on synonymous differences. Bootstrap support was obtained by 1,000 independent replicates.
(1.30 MB TIF)

Figure S3 Phylogenies of 68 SRK sequences from *A. lyrata* and *A. halleri*. The phylogeny was obtained by the neighbour-joining method on non-synonymous differences. Bootstrap support was obtained by 1,000 independent replicates.
(1.36 MB TIF)

Figure S4 Bootstrap distribution (10,000 replicates) of net divergence for SRK alleles (average across 18 S alleles pairs, in black) and the genomic background (average across 12 control genes, in grey).
(0.86 MB TIF)

Figure S5 Distribution of the number of pairwise nucleotide differences for SRK sequences in interspecific comparisons between *A. halleri* and *A. lyrata*, excluding the 18 pairs of sequences considered as transspecific pairs.
(0.43 MB TIF)

Table S1 Net divergence estimation for the 12 control genes.
(0.05 MB DOC)

Text S1 Supplemental material.
(0.05 MB DOC)
