# Supplementary figures and images for: Repeated Adaptive Introgression at a Gene under Multiallelic Balancing Selection
Source: PLoS Genet. 2008 Aug 29;4(8):e1000168. doi: 10.1371/journal.pgen.1000168 (PMC2517234; doi:10.1371/journal.pgen.1000168)

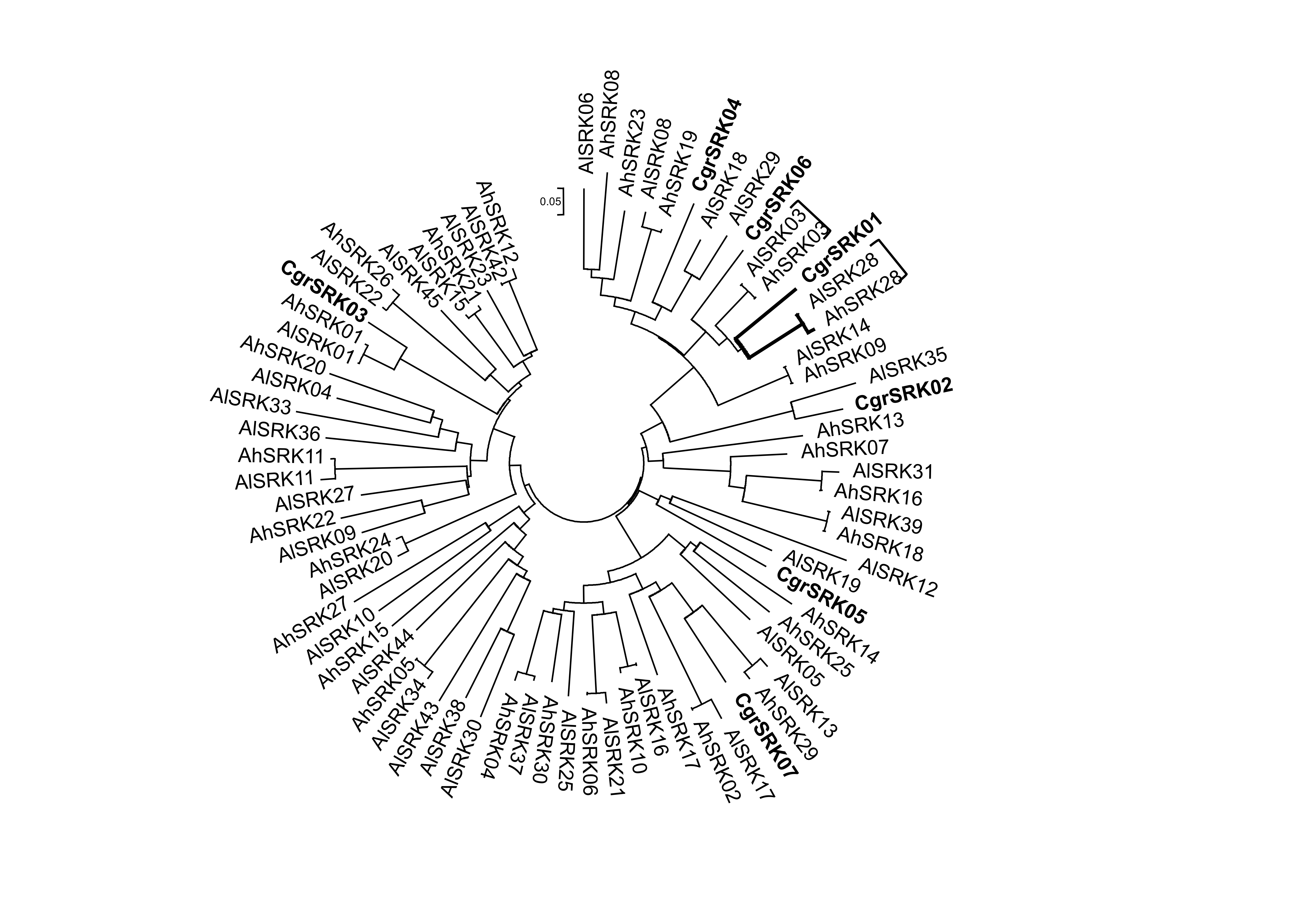

Supplement: Figure S1 — Phylogeny of SRK sequences from the species A. lyrata (n = 38), A. halleri (n = 30) and Capsella grandiflora (n = 7, shown in bold). The phylogeny was obtained by the neighbour-joining method on the proportion of amino-acid differences. Brackets indicate the position of two trans-specifically shared pairs of S-alleles between A. lyrata and A. halleri that are interrupted by the branching of one S-alleles from C. grandiflora (thick lines). (1.16 MB TIF) [file pgen.1000168.s001.tif]

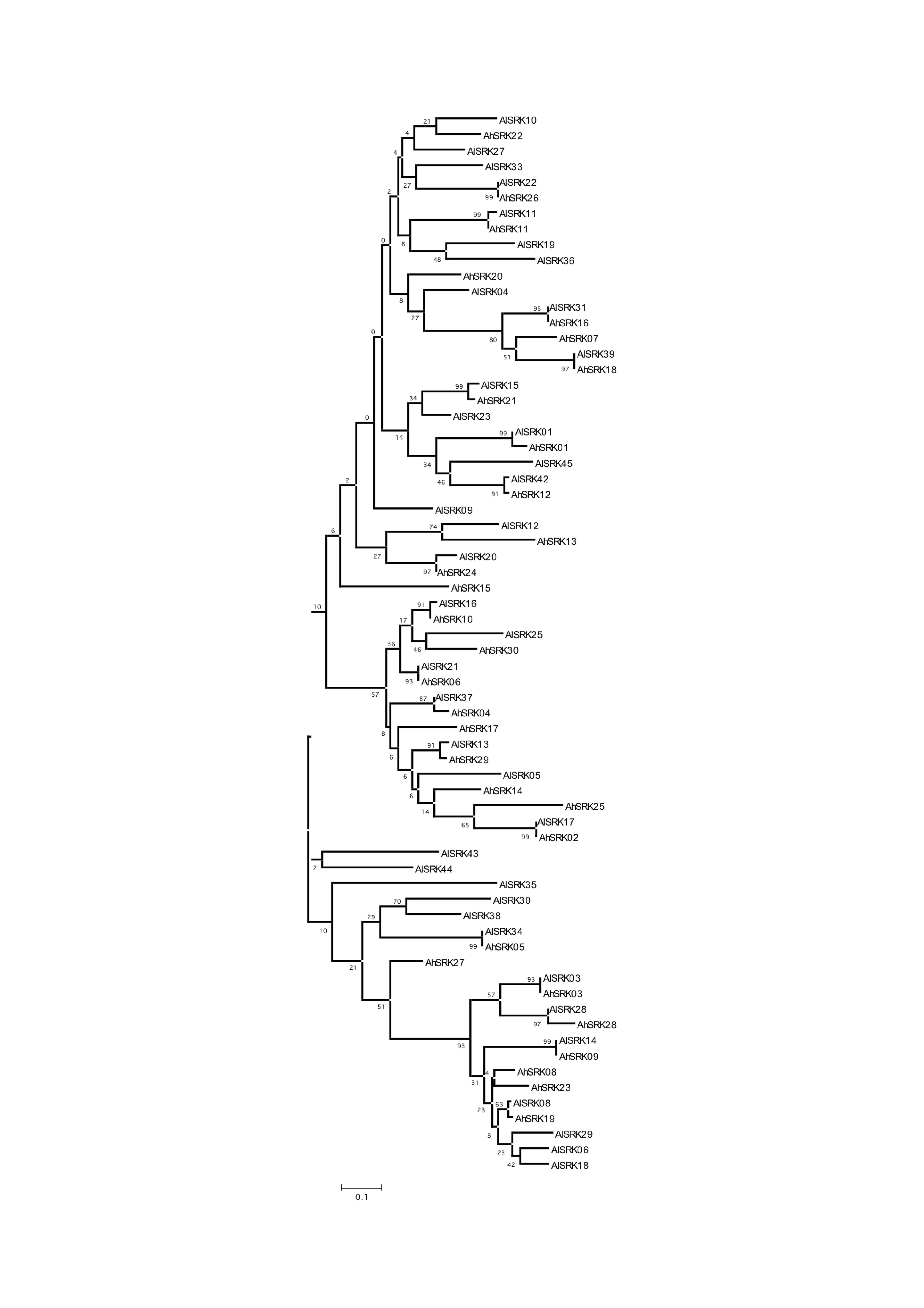

Supplement: Figure S2 — Phylogenies of 68 SRK sequences from A. lyrata and A. halleri. The phylogeny was obtained by the neighbour-joining method on synonymous differences. Bootstrap support was obtained by 1,000 independent replicates. (1.30 MB TIF) [file pgen.1000168.s002.tif]

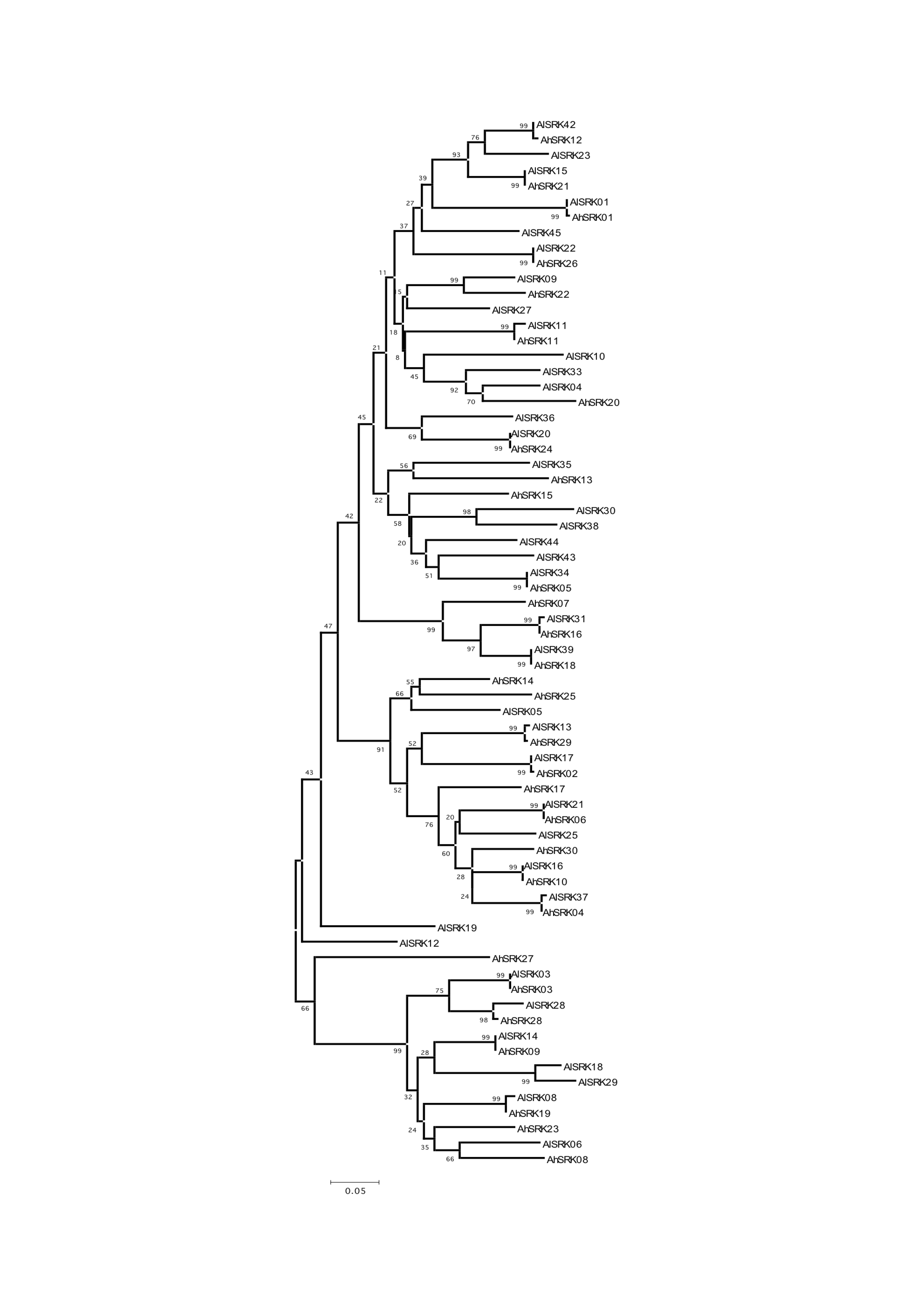

Supplement: Figure S3 — Phylogenies of 68 SRK sequences from A. lyrata and A. halleri. The phylogeny was obtained by the neighbour-joining method on non-synonymous differences. Bootstrap support was obtained by 1,000 independent replicates. (1.36 MB TIF) [file pgen.1000168.s003.tif]

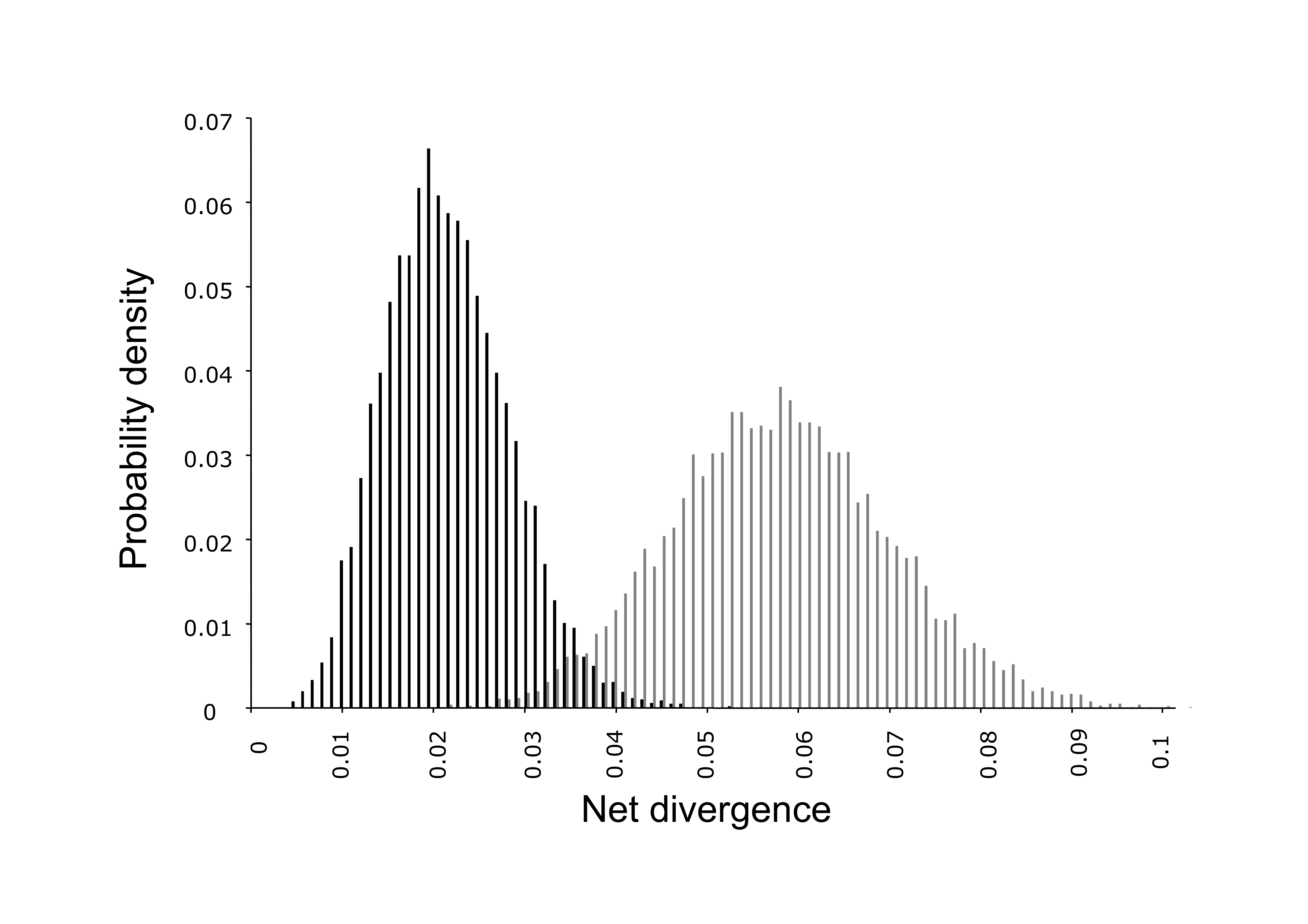

Supplement: Figure S4 — Bootstrap distribution (10,000 replicates) of net divergence for SRK alleles (average across 18 S alleles pairs, in black) and the genomic background (average across 12 control genes, in grey). (0.86 MB TIF) [file pgen.1000168.s004.tif]

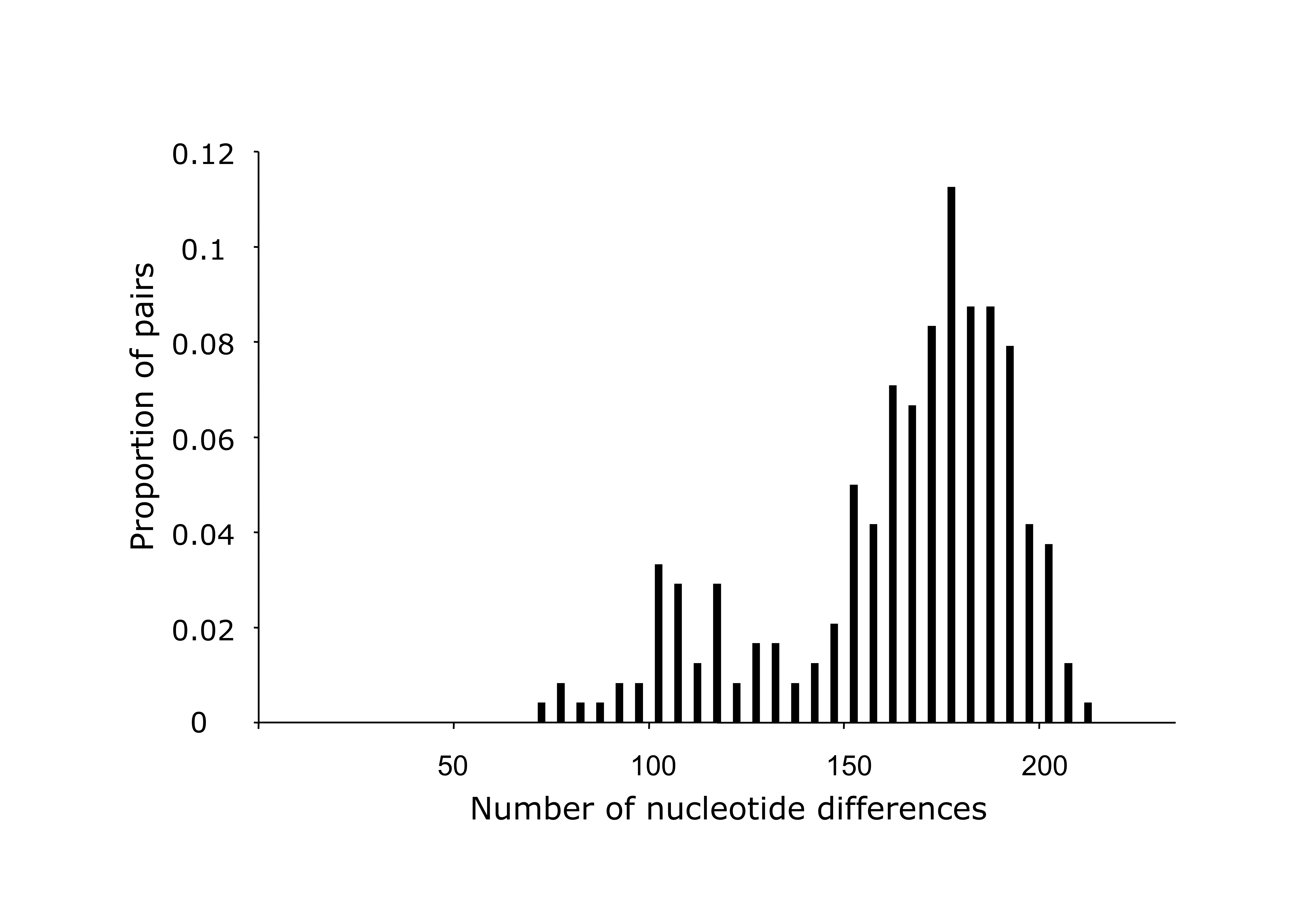

Supplement: Figure S5 — Distribution of the number of pairwise nucleotide differences for SRK sequences in interspecific comparisons between A. halleri and A. lyrata, excluding the 18 pairs of sequences considered as transspecific pairs. (0.43 MB TIF) [file pgen.1000168.s005.tif]
