# Supplementary material for: Repeated Adaptive Introgression at a Gene under Multiallelic Balancing Selection
Source: PLoS Genet. 2008 Aug 29;4(8):e1000168. doi: 10.1371/journal.pgen.1000168 (PMC2517234; doi:10.1371/journal.pgen.1000168)
Supplement: Table S1 — Net divergence estimation for the 12 control genes. (0.05 MB DOC) [file pgen.1000168.s006.docx]

|  | *K*_4fold_ | *π_lyrata_* | *π_halleri_* | Net divergence |
| --- | --- | --- | --- | --- |
| CAD | 0.0483 | 0.0061 | 0.0256 | 0.0324 |
| CHI | 0.1468 | 0.0115 | 0.0092 | 0.1365 |
| CHS | 0.0881 | 0.0370 | 0.0122 | 0.0635 |
| DFR | 0.0610 | 0.0364 | 0.0472 | 0.0192 |
| F3H | 0.1223 | 0.0131 | 0.0051 | 0.1132 |
| FAH1 | 0.0739 | 0.0055 | 0.0095 | 0.0664 |
| GS | 0.0861 | 0.0395 | 0.0361 | 0.0483 |
| MAML | 0.0312 | 0.0142 | 0.0076 | 0.0203 |
| CAUL | 0.0184 | 0.0000 | 0.0167 | 0.0100 |
| HAT4 | 0.0531 | 0.0291 | 0.0000 | 0.0386 |
| ScADH | 0.0323 | 0.0165 | 0.0256 | 0.0112 |
| Aly9 | 0.1296 | 0.0500 | 0.0117 | 0.0988 |
| **Average** | |  |  | **0.0549** |
| **95%CI** | |  |  | **[0.0340 – 0.0795]** |
