# Supplementary material for: Repeated Adaptive Introgression at a Gene under Multiallelic Balancing Selection
Source: PLoS Genet. 2008 Aug 29;4(8):e1000168. doi: 10.1371/journal.pgen.1000168 (PMC2517234; doi:10.1371/journal.pgen.1000168)
Supplement: Text S1 — Supplemental material. (0.05 MB DOC) [file pgen.1000168.s007.doc]

**Supplemental material**

**Criteria to define trans-specifically shared S-alleles**

Based on the mismatch distribution for interspecific pairs of *SRK* sequences between *A. lyrata* and *A. halleri* (Fig. 2), we defined "trans-specifically shared S-alleles", *i.e.* alleles assumed to have evolved from a single S-allele in the direct ancestor of *A. lyrata* and *A. halleri*, as those whose sequence divergence was below a cut-off point of 12 nucleotide differences. The following arguments are supporting the view that other interspecific pairs of *SRK* sequences cannot be considered as representing trans-specifically shared S-alleles:

1. The two closest interspecific pairs of sequences above the cut-off point showing intermediate levels of divergence (45 between AlSRK03 and AhSRK28, and 50 between AlSRK28 andAhSRK03, see Fig. 2) cannot represent trans-specifically shared S-alleles because they are not monophyletic (see Fig. 1 and Fig. S1). Indeed the closest sequence to AlSRK03 is AhSRK03 and the closest to AlSRK28 is AhSRK28. Since nucleotide divergence within each pair is below the cut-off point, both pairs are considered to be trans-specific, such that AlSRK03/AhSRK28 and AlSRK28/AhSRK03 cannot represent pairs of trans-specifically shared S-alleles. Moreover, we computed a phylogeny using additionally *SRK* sequences from the outgroup *Capsella grandiflora* (Paetsch et al. 2006, Nasrallah et al. 2007), and found that one S-alleles from *C. grandiflora* (CgrSRK01) is branching at an intermediate position between the pair AlSRK03/AhSRK03 and the pair AlSRK28/AhSRK28 (Fig. S1). This suggests that the divergence between these two closest interspecific pairs of sequences above the cut-off point is older than time of origin of the Arabidopsis genus.
2. The next two closest interspecific pairs of sequences show levels of divergence more than five times higher that the cut-off point (72 between AlSRK06 and AhSRK23, and 95 between AlSRK04 and AhSRK20, see Fig. 1). Moreover, these two pairs of sequences show substantially higher ratios of non-synonymous over synonymous nucleotide differences (K_A_/K_S_ = 0.91 for AlSRK06 and AhSRK23, and K_A_/K_S_ = 1.13 for AlSRK04 and AhSRK20) than the sequences from the set of trans-specifically shared S-alleles (average K_A_/K_S_ = 0.29). This is likely to be due to positive selection on non-synonymous sites during the evolution of new allelic specificities at *SRK* along the lineages separating these two pairs of sequences, as commonly reported in molecular evolution studies of the S-locus in Brassicaceae (*e.g.* [23]). Hence, these two pairs of sequences are certainly corresponding to four distinct S-alleles present in the common ancestor of both *A. halleri* and *A. lyrata*.

**Net divergence estimation**

We estimated net divergence for the genomic background according to [41], by substracting the mean within-species diversity from the raw divergence:

*D_A_ = K*_4fold_ - (*π_lyrata_* + *π_halleri_*)/2

Because polymorphism among gene copies of *SRK* alleles is extremely low ([22], this study), we conservatively assumed *π*=0, thus considering that net divergence equals raw divergence for this gene.

Net divergence averaged 0.0549 but was variable across control genes, ranging from 0.0100 for CAUL to 0.1365 for CHI (Table S1). We then used a bootstrap procedure (10,000 replicates) to test the null hypothesis that net divergence for *SRK* alleles is identical to that for the genomic background (Fig. S4). The 95% CIs for *SRK* alleles ([0.0084 - 0.0336]) and the genomic background ([0.0340 - 0.0795], Table S1) are not overlapping, so we reject the null hypothesis and confirm that divergence at *SRK* alleles is lower than that at control genes.

**References**

Nasrallah JB, Liu P, Sherman-Broyles S, Schmidt R, Nasrallah ME. (2007). Epigenetic mechanisms for breakdown of self-incompatibility in interspecific hybrids. Genetics 175: 1965-1973.

Paetsch M, Mayland-Quellhorst S, Neuffer B (2006) Evolution of the self-incompatibility system in the Brassicaceae: identification of S-locus receptor kinase (*SRK*) in self-incompatible *Capsella grandiflora.* Heredity 97:283-290.
